# Shape-Programmable Liquid Metal Fibers

**DOI:** 10.3390/bios13010028

**Published:** 2022-12-26

**Authors:** Biao Ma, Jin Zhang, Gangsheng Chen, Yi Chen, Chengtao Xu, Lanjie Lei, Hong Liu

**Affiliations:** State Key Laboratory of Bioelectronics, School of Biological Science and Medical Engineering, Southeast University, Nanjing 210096, China

**Keywords:** liquid metal, conductive fiber, stretchable electrodes, flexible electronics, wearable sensors

## Abstract

Conductive and stretchable fibers are the cornerstone of intelligent textiles and imperceptible electronics. Among existing fiber conductors, gallium-based liquid metals (LMs) featuring high conductivity, fluidity, and self-healing are excellent candidates for highly stretchable fibers with sensing, actuation, power generation, and interconnection functionalities. However, current LM fibers fabricated by direct injection or surface coating have a limitation in shape programmability. This hinders their applications in functional fibers with tunable electromechanical response and miniaturization. Here, we reported a simple and efficient method to create shape-programmable LM fibers using the phase transition of gallium. Gallium metal wires in the solid state can be easily shaped into a 3D helical structure, and the structure can be preserved after coating the wire with polyurethane and liquifying the metal. The 3D helical LM fiber offered enhanced stretchability with a high breaking strain of 1273% and showed invariable conductance over 283% strain. Moreover, we can reduce the fiber diameter by stretching the fiber during the solidification of polyurethane. We also demonstrated applications of the programmed fibers in self-powered strain sensing, heart rate monitoring, airflow, and humidity sensing. This work provided simple and facile ways toward functional LM fibers, which may facilitate the broad applications of LM fibers in e-skins, wearable computation, soft robots, and smart fabrics.

## 1. Introduction

Soft and stretchable electronics that can conformally deform with soft tissues and nonplanar surfaces hold great promise for wearables [1], human–machine interfaces [2], the Internet of Things [3], and soft robotics [4,5]. Stretchable electronics based on the fiber format are attractive in creating smart textiles for long-term health monitoring, wearable computation, and biosensing [6,7,8]. For conventional fiber electronics, selecting favorable conductive materials is essential to their performance and multi-functionality. Existing fiber conductors such as conductive polymers, carbon materials, conductive nanocomposite, metals, and ionic conductors [9,10] have at least one of the following limitations: low conductivity, complicated synthesis, high modulus, and poor biocompatibility. In contrast, gallium-based liquid metals (LMs) featuring high conductivity, fluidity, good biocompatibility, and self-healing are quite attractive for creating high-performance fiber electronics for interconnection, power generation, sensing, or actuation [11,12,13,14,15]. LM-based fiber electronics have demonstrated their merits in implantable pressure sensors, wearable energy harvesting, information processing, and soft robotics [16,17,18].

The liquid nature of LM makes it straightforward to fabricate conductive fibers by directly injecting LM into hollow microfibers [19,20,21,22]. Alternatively, LM can also be coated on target fibers without the hollow channel; however, a surface treatment step is required to enhance the adhesion between LM and fiber surfaces [23,24,25]. In addition, LM fibers can also be obtained by electrochemical spinning [26] to lower the surface tension of LM for scalable production or direct wet spinning of LM–polymer nanocomposite [27]. Although various fabrication methods and many impressive applications have emerged, current LM fibers are limited to 1D structures, hindering the realization of tunable electromechanical properties and multi-functionality. In addition, it is also challenging to obtain ultrafine and lightweight LM fibers in a facile and low-cost manner. Previously reported methods to reduce the diameter of LM fibers typically rely on thermal drawing [28], but this method involves high-temperature operation and specific equipment, posing a limit of high energy consumption and high-cost operation.

In this work, we report simple and efficient methods to program the shape and diameter of LM fibers. This method takes advantage of the plasticity of gallium wires in the solid state that allows for the shaping of 1D wires into complex 3D helical structures. After coating the fibers with a thin layer of polyurethane (PU) elastomer, the 3D structure can be permanently preserved even when the gallium transforms into the liquid state. These 3D LM fibers offer an enhanced stretchability of 1273% and show invariable conductance over 283% strain. We also show that helical LM fibers can be used for self-powered strain sensing based on the electromagnetic induction effect. In addition, we also found that the diameter of the LM fiber can be reduced just by stretching the fiber during the PU solidification process; the fiber diameter decreased from 343 to 108 μm when a strain of 500% was applied. This provided a facile way to create lightweight and ultrafine LM fibers without high-temperature operation. The potential applications of ultrafine fibers in wearable heart rate sensors and airflow and humidity sensors are also demonstrated.

## 2. Materials and Methods

### 2.1. Materials

Gallium (99.99%) was obtained from Dingguan Metal Technology Co., Ltd. (Dingguan, China) Silicone tubes (inner diameter: ~300 μm) were obtained from Taizhou Chunshi New Material Co., Ltd. (Taizhou, China) Polyurethane (PU) pellets (1180A, BASF) were obtained from Xiangqing Plastics Co., Ltd. (Jieshou, China) N, N-dimethylformamide (DMF, 99%) was obtained from Sinopharm Chemical Reagent Co. Ltd. (Shanghai, China). The PU solution was obtained by dissolving PU pellets in DMF at 70 °C for 2 h under mechanical stirring. 

### 2.2. Preparation of Helical LM Fibers

We prepared the solid gallium wires by injecting the liquid gallium into the silicone tube. Then, the wires were cooled at −20 °C for ~1 h to solidify the gallium. The silicone tube was sliced away using a surgical blade to extract the solid gallium wire. A helical gallium wire was obtained by helically winding the gallium wire on a glass tube with a diameter of 1.5 mm. The helical gallium wire was coated with PU by dip-coating, then immersed in water to solidify the PU.

### 2.3. Preparation of Ultrafine LM Fibers

We immersed the solid gallium wire coated with PU precursor into 40 ℃ water for ~2 s. Then, the fiber was uniaxially stretched, and the stretched fiber was held in the water for ~60 s to fix the shape.

### 2.4. Characterization

A digital multimeter (DMM6500, Keithley, Cleveland, OH, USA) was used for electric resistance measurements. Microscopical characterization was conducted using a microscope (SZX16, Olympus, Tokyo, Japan). Field emission scanning electron microscopy (FE-SEM) characterization was performed using an UltraPlus Zeiss microscope system. A commercial humidity sensor (AM1011A, ASAIR, Guangzhou, China) was used for humidity measurements.

## 3. Results and Discussion

### 3.1. Shape-Programmable LM Fibers

**Figure 1:**
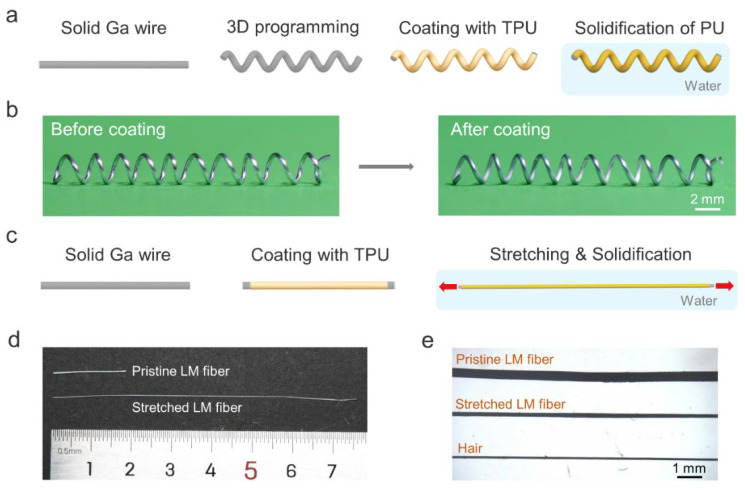
Shape-programmable LM fibers. (**a**) Schematic illustration of the preparation of 3D helical LM–PU fibers. (**b**) Photographs of the LM before and after coating with PU. (**c**) Schematic illustration of the reduction of the fiber diameter during PU solidification. (**d**) Comparison of the LM fiber before and after stretching. (**e**) Optical microscope photographs of LM fibers and human hair.

Figure 1a schematically depicts the shape programmability of solid gallium wire. The gallium wire with plasticity has a melting point of 29.8 °C [29], which allows us to shape its structure at room temperature. Here, we chose the 3D helical structure due to its potential applications in highly stretchable fibers and magnetic sensors. The gallium metal wire was obtained by injecting liquid gallium into a commercially available silicone tube with an inner diameter of ~300 μm. After cooling to the solid state, the gallium wire was extracted from the silicone tube and helically winded on a glass tube. To obtain a completely soft and stretchable 3D LM fiber, we coated the helical gallium wire with a thin layer of polyurethane (PU) solution by dip-coating. Then, the fiber was immersed in a water bath to remove the N, N-dimethylformamide (DMF) solvent to solidify the PU [30]. Figure 1b shows a typical 3D helical LM fiber before and after coating the elastic PU layer. It is worth noting that the helical gallium fiber without the PU shell could not maintain the 3D structure when the gallium was in the liquid state. In contrast, the one with the PU shell maintained its helical structure after heating, as shown in Figure S1, Supplementary Materials. Although the solid gallium wire within the silicone tube can also be deformed into a 3D structure, this structure could not be preserved after the melting of gallium due to the elastic recovery of the silicone tube. In addition, we can also weld the solid gallium wires using gallium to create other 3D structures, such as tetrahedral or cubic structures.

In addition, the diameter of the LM fiber can also be reduced during the solidification of PU. Specifically, the gallium wire rapidly transformed into a liquid state when the PU-coated LM fiber was immersed in warm water (~40 °C). Before the complete solidification of PU, the fiber was uniaxially stretched to reduce its diameter, and then the PU was fully solidified to fix the shape (Figure 1c). Notably, the conventional method to obtain ultrafine core–shell LM fibers using thermal drawing requires heating of the thermoplastic shell to high temperatures [28]. Our approach eliminates high energy consumption and does not require specific high-cost drawing equipment, facilitating the fabrication of ultrafine LM fibers cost-efficiently. Figure 1d shows optical images of fibers with and without stretching. The diameter of stretched LM fibers is slightly higher than that of human hairs (Figure 1e). 

### 3.2. Optimization of Dip-Coating and Characterization of LM Fibers

**Figure 2:**
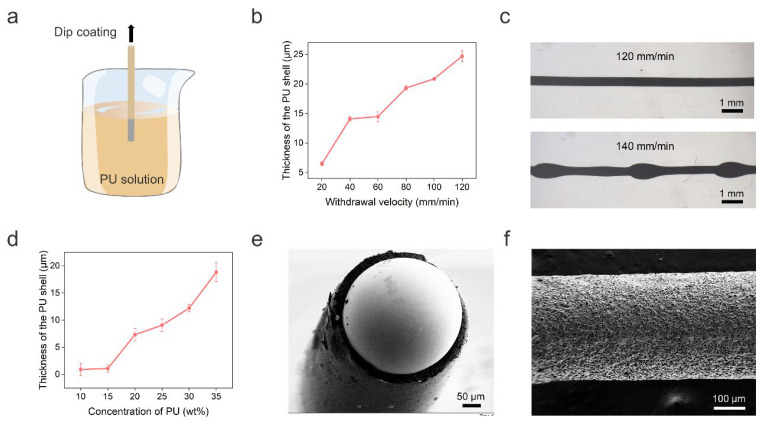
Preparation and characterization of the core–shell LM fiber. (**a**) Schematic illustration of the preparation of core–shell LM fiber by dip-coating. (**b**) Thickness of the PU shell as a function of the withdrawal velocity. (**c**) Representative microscope photographs of the LM fiber with 120 mm/min and 140 mm/min withdrawal velocities. (**d**) Thickness of the PU shell as a function of the PU concentration. (**e**) Scanning electron micrograph (SEM) of the cross section of the core–shell LM fiber. (**f**) SEM of the rough shell surface.

The PU shell of the LM fiber was obtained by dip-coating, as shown in Figure 2a. Specifically, we immersed the solid gallium wire into PU solution with a concentration of 35 wt%. Then, the solid wire was drawn out from the solution at a fixed withdrawal velocity. We investigated the influence of the withdrawal velocity on the PU shell thickness, which increased with increasing withdrawal velocity in the range of 20–120 mm/min (Figure 2b). However, when the withdrawal velocity increased to 140 mm/min, we observed the break-up of the PU solution into droplets on the gallium wire due to the Rayleigh instability effect [31], as shown in Figure 2c. This hinders the formation of a PU shell with uniform thickness. We also noticed that when the withdrawal velocity was below 80 mm/min, the shell thickness was too thin to support the 3D structure. Here, we chose a withdrawal velocity of 80 mm/min to balance the prevention of Rayleigh instability, mechanical strength of the PU shell, and the reduction of PU consumption. Note that the withdrawal velocity was optimized for a straight LM wire. For the helical LM wire, we adjusted the speed based on the ratio of the total length of the wire and the length of the helical spring. In addition, the PU thickness can be adjusted by changing the PU concentration. Figure 2d shows that the shell thickness increased with increasing PU concentration from 10 wt% to 35 wt% at 80 mm/min. Since a thicker PU shell can provide better support for the 3D structure, we chose 35 wt% PU solution for the following experiments. Figure 2e shows a typical cross-sectional view of the fiber with a clear LM core and PU shell structure. The formation of a rough PU shell surface was due to the dual diffusion between the DMF solvent and water (Figure 2f). 

### 3.3. Applications of the Helical LM Fiber

**Figure 3:**
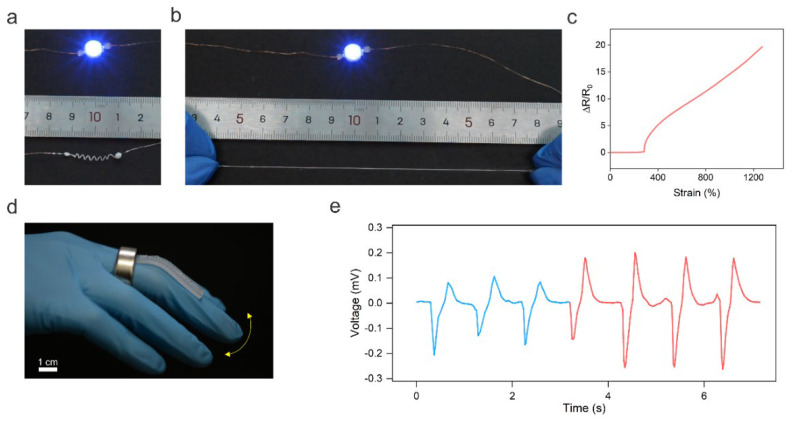
3D helical LM fibers. (**a**) The helical LM fiber is connected in series with a blue LED. (**b**) The helical LM fiber maintained its conductivity under stretching. (**c**) Change in relative resistance of the LM fiber as a function of tensile strain. (**d**) Optical image of the LM fiber-based strain sensor to monitor the finger bending based on Faraday’s law of electromagnetic induction. (**e**) Real-time change in the induced voltage in response to finger bending.

Helical shapes are ubiquitous in nature, ranging from the double helices formed by DNA to the climbing tendrils of plants [32]. Endowing conductive fibers with 3D helical architectures has been demonstrated as an attractive way to enhance fibers’ stretchability and regulate their electromechanical response [33]. Moreover, functional devices such as magnetic sensors [34] and soft actuators [35] can also be created using structured 3D helical fibers. Thus, in this work, we programmed the solid 1D LM wire into a helical shape to extend the applications of LM fibers. To show the stretchability of the helical LM fiber, a blue LED was placed in series connection with the fiber to indicate the electric connection of the fiber under stretching (Figure 3a). As shown in Figure 3b, the helical LM fiber maintained conductivity at ~600% strain. The stretchability test was performed when the LM core was in liquid phase at room temperature (~25 °C). Note that the gallium has a supercooling effect and, thus, the LM core can maintain a liquid state for a long period of ~48 h at ~25 °C once it is heated to the liquid state. Additionally, heating the gallium wire is quite simple just by using body heat or warm water. Alternatively, the melting point of the LM core can be lowered by dispersing indium microparticles to alloy with the gallium.

In addition, the helical LM fiber shows invariable conductance over 283% strain (Figure 3c). This is because the stretching-induced unwinding process did not change the fiber’s total length and cross-sectional area. Thus, the resistance of the LM fiber did not alter upon stretching. The strain insensitivity of the helical LM fiber makes it attractive for high-performance stretchable and stable electrodes. When the strain was higher than 283%, the resistance of the LM fiber increased with increasing strain, indicating its potential application in sensing high values of strain. The different electromechanical responses of helical LM fibers at different strain ranges may be used for creating intelligent fibers for wearable computation. The helical LM fiber fractured at ~1273% strain, which is much higher than that of 358% of the 1D LM fiber, indicating that the helical structure can enhance the stretchability of the LM fiber.

In addition, the helical LM fiber can also perceive magnetic field changes based on Faraday’s law of electromagnetic induction, allowing it to be used as a self-powered wearable strain sensor to monitor body motions. As a proof of concept, the helical LM fiber was glued onto the elastomer; then the sensor was fixed onto the finger. A permanent ring magnet worn on the finger was used to generate a magnetic field, as shown in Figure 3d. The finger bending resulted in the deformation of the helical LM fiber, leading to a change in the magnetic flux density in the helical fiber. Figure 3e shows the induced voltage in response to different bending angles of 42° (blue line) and 72° (red line). The self-powered strain sensor can be potentially used for human–machine interactions or monitoring Parkinson’s disease. 

### 3.4. Applications of Ultrafine LM Fibers

**Figure 4:**
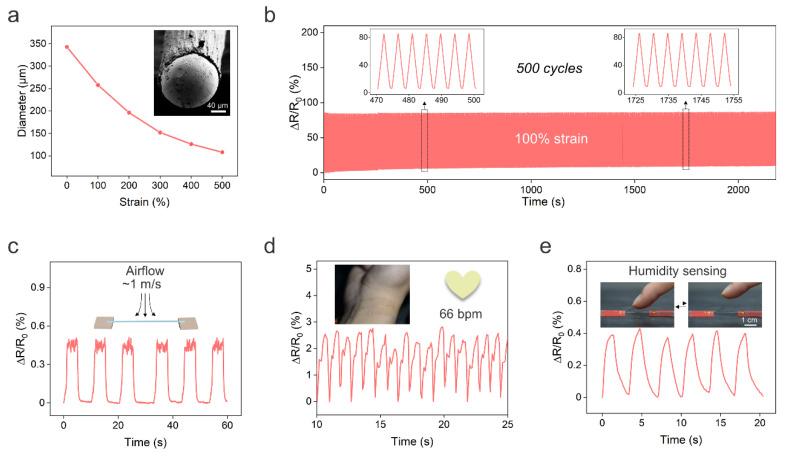
Ultrafine LM fibers. (**a**) Fiber diameter as a function of the tensile strain. Insert is a typical SEM image of the cross section of the fiber. (**b**) Relative resistance change under cycling stretching/releasing test at 100% strain. (**c**) Change in relative resistance of the ultrafine LM fiber in response to airflow at the velocity of 1 m/s. (**d**) The ultrafine LM fiber can also be used as a pressure sensor to monitor the heart rate. (**e**) The relative resistance of the LM fiber in response to the humidity change when the finger approached the fiber.

Unintentionally, we also found that we could decrease the fiber diameter during the solidification of PU. In the solidification process, the LM wire coated with PU solution was immersed in warm water (~40 °C), and the LM was immediately heated to the liquid state. Meanwhile, the water would diffuse into the PU solution and the DMF in the solution would gradually ooze out to achieve solidification of PU. Once the outer layer of the PU solution made contact with the water, it immediately solidified, and the non-solidified phase and LM core were encapsulated in the fiber. After remaining in contact with the water for ~2 s, the fiber was stretched to decrease the fiber diameter. The stretched fiber was then held in the water to allow for the solidification of the inner layer of the PU. We observed that the stretched LM fiber can maintain its shape after solidification for ~60 s. Thus, we can program the fiber diameter during solidification without involving high-cost microfabrication facilities and high-temperature operations such as thermal drawing. The fiber diameter decreased from 343 to 108 μm when the strain was increased from 0 to 500% (Figure 4a), which is lower than that of previously reported core–shell LM fibers created by direct injection [19,20]. The ultrafine LM fiber is lightweight, flexible, stretchable, and highly conductive. Thus, the LM fiber can be integrated into fabrics for wearable applications. The ultrafine LM fiber also exhibited good electromechanical stability under the cycling stretching/releasing test. Figure 4b depicts the real-time changes in the relative resistance of the LM fiber under cyclic stretching/releasing tests at 100% strain for 500 cycles. The resistance of the LM fiber slightly increased by ~9% after the cycling strain test. In addition, the ultrafine LM fiber maintained electrical conductivity with a tensile strain of ~200% as shown in Figure S2.

Next, we demonstrate the potential applications of ultrafine LM fibers. Firstly, the light weight of the LM fiber enabled it to sense airflow. Figure 4c shows the real-time changes in relative resistance of the sensor in response to a low airflow at ~1 m/s. Since airflow sensation is a vital function of bodies, the LM fiber-based airflow sensor shows great promise for applications such as e-skins and robotics. Moreover, the LM fiber can also be utilized as a pressure sensor to monitor wrist pulse for heart rate monitoring, as shown in Figure 4d. 

Another interesting and unexpected application of ultrafine LM fibers is humidity sensing. The humidity level of the human skin can reflect metabolic and physiological states under different conditions [36]. Humidity sensing can also be used to monitor wound healing and respiration, achieve touchless human–machine interactions, further environmental analysis, and so forth [37]. Conventional humidity sensors have limitations in synthesizing humidity-responsive materials and device fabrication. We found that the resistance of the LM fiber rapidly increased when a moist object, such as a piece of hydrogel, approached the fiber. The resistance returned to baseline after withdrawing the hydrogel (Figure S3). We note that both the hydrogel and the fiber are at room temperature and, thus, we can exclude the influence of the temperature on the resistance increase. Surprisingly, the LM fiber could also perceive a rise in moisture when a human finger approached the fiber (2 mm between fiber and fingertip), and the relative humidity increase was ~23%, as shown in Figure 4e. Moreover, the resistance response to humidity is quite rapid, with a response time of ~1.3 s. The sensing mechanism is likely the cause of the PU being able to absorb water molecules from the environment since it has hydrophilic groups such as CO and NH. The absorbed water may result in the swelling of the PU [37,38] and, thus, the LM core is compressed, leading to a decrease in the cross-sectional area and an increase in resistance. We also tried to cool the LM to the solid state to sense the humidity change, but the solid LM fiber did not respond to the humidity. This is because the swelling of the PU could not result in the deformation of the solid LM core. In addition, the fiber with a larger diameter of LM did not respond to the humidity change (Figure S4). This is because the swelling of the PU is insufficient to drive the deformation of the large LM core. This also highlighted the importance of creating ultrafine LM fibers for humidity sensing.

## 4. Conclusions

In this work, we report two simple and facile ways to create shape-programmable LM fibers. One was achieved by using the plasticity of solid gallium wire. The 1D solid gallium wire can be shaped into 3D helical structures, and the structures can be maintained after coating the wire with PU, even if the gallium was heated to the liquid state. The 3D helical LM fibers can be used as stretchable conductors with invariable conductance under ~283% strain, and self-powered strain sensors for human motion monitoring. We also show the diameter of the LM fiber can be reduced by applying tensile strain to the fiber during PU solidification, providing an efficient way to create ultrafine LM fibers with potential applications in e-skins, sensitive airflow sensing, and humidity sensing. Programmable LM fibers may find wide applications in soft robotics, implantable devices, and wearable computation. Future improvements in scalable fabrication and the use of other active coatings for wide applications are undergoing.

## Data Availability

Not applicable.

## Supplementary Materials

The following supporting information can be downloaded at: https://www.mdpi.com/article/10.3390/bios13010028/s1, Figure S1: Comparations of the helical gallium fiber without and with PU shell after heating; Figure S2: Change in relative resistance of the ultrafine LM fiber as a function of the tensile strain; Figure S3: Change in relative resistance of the ultrafine fiber in response to humidity change; Figure S4: Change in relative resistance of the pristine fiber (without stretching) in response to humidity change.
