# Supplementary material for: Shape-Programmable Liquid Metal Fibers
Source: Biosensors (Basel). 2022 Dec 26;13(1):28. doi: 10.3390/bios13010028 (PMC9856024; doi:10.3390/bios13010028)
Supplement: Supplementary file 1 [file biosensors-13-00028-s001.zip › biosensors-2094308-supplementary.pdf]

biosensors

Supporting Information

Shape-Programmable Liquid Metal Fibers

Biao Ma†, Jin Zhang†, Gangsheng Chen, Yi Chen, Chengtao Xu and Hong Liu*

State Key Laboratory of Bioelectronics, School of Biological Science and Medical Engineering, Southeast University, Nanjing 210096, China
* Correspondence: liuh@seu.edu.cn
† These authors contributed equally to this work.

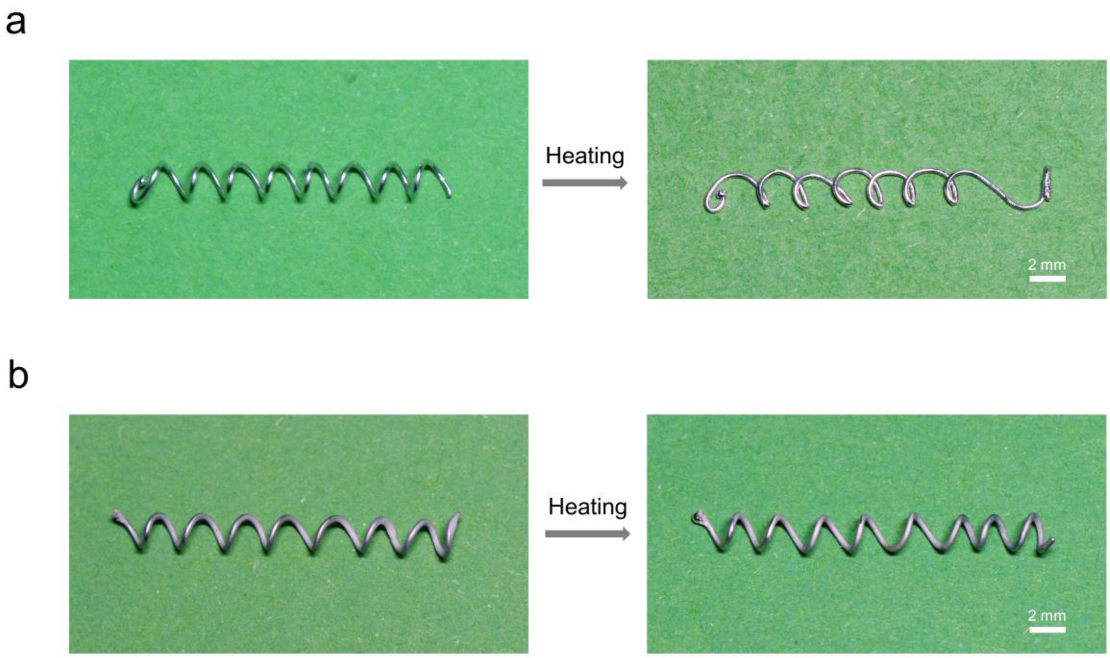

Figure S1. Comparations of the helical gallium fiber without (**a**) and with (**b**) PU shell after heating. The helical structure can be maintained with the support of a PU shell when the gallium was in the liquid state.

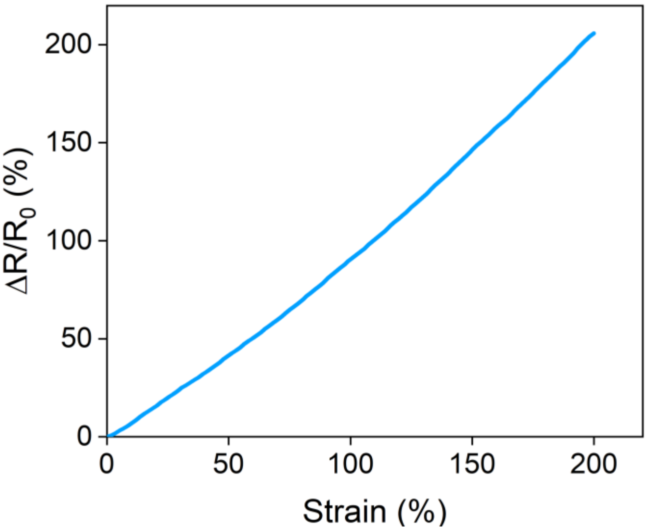

Figure S2. Relative resistance change of the ultrafine LM fiber as a function of the tensile strain.

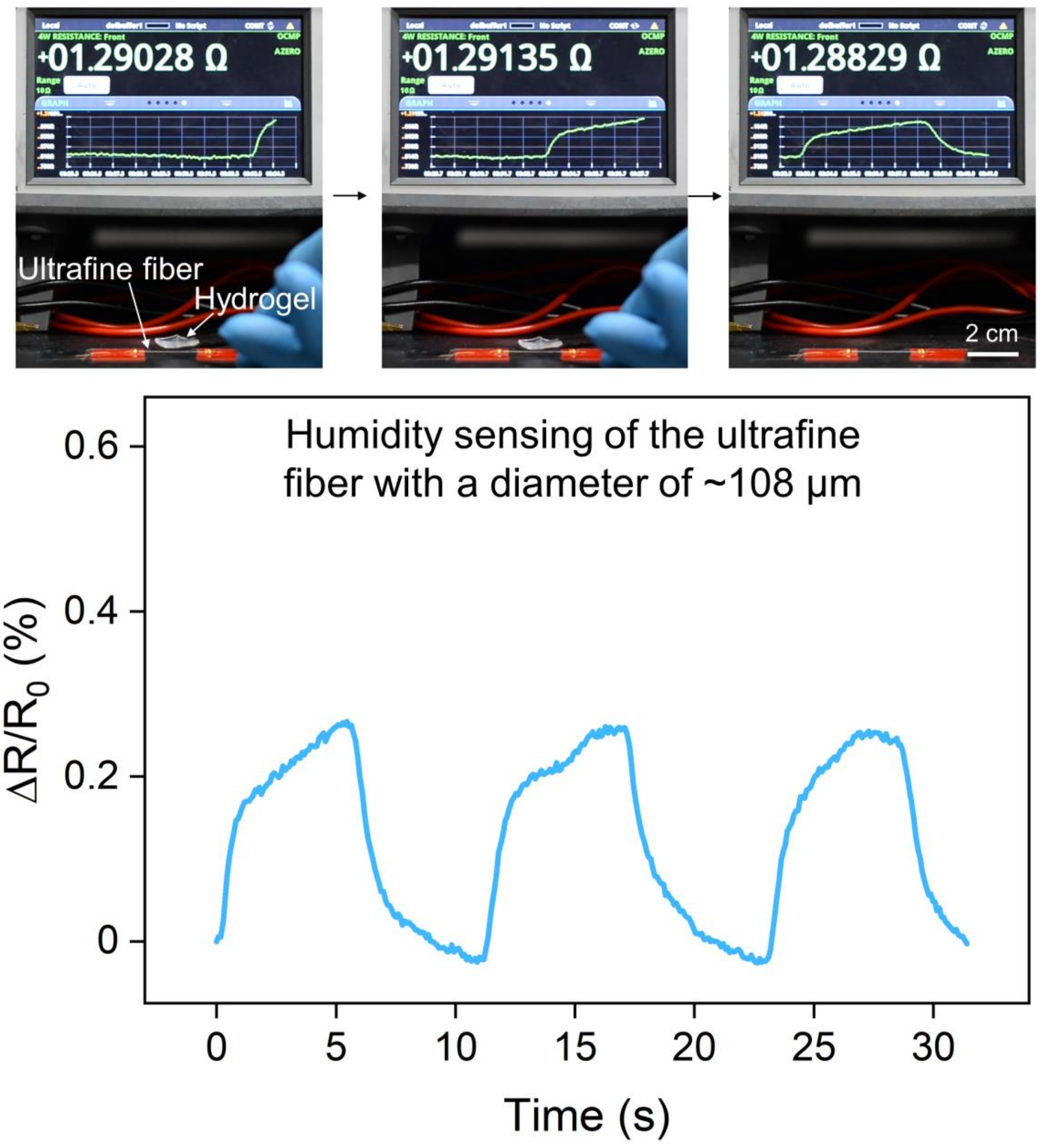

Figure S3. Humidity sensing of the ultrafine fiber. The resistance of ultrafine fiber increased rapidly when a piece of hydrogel approached.

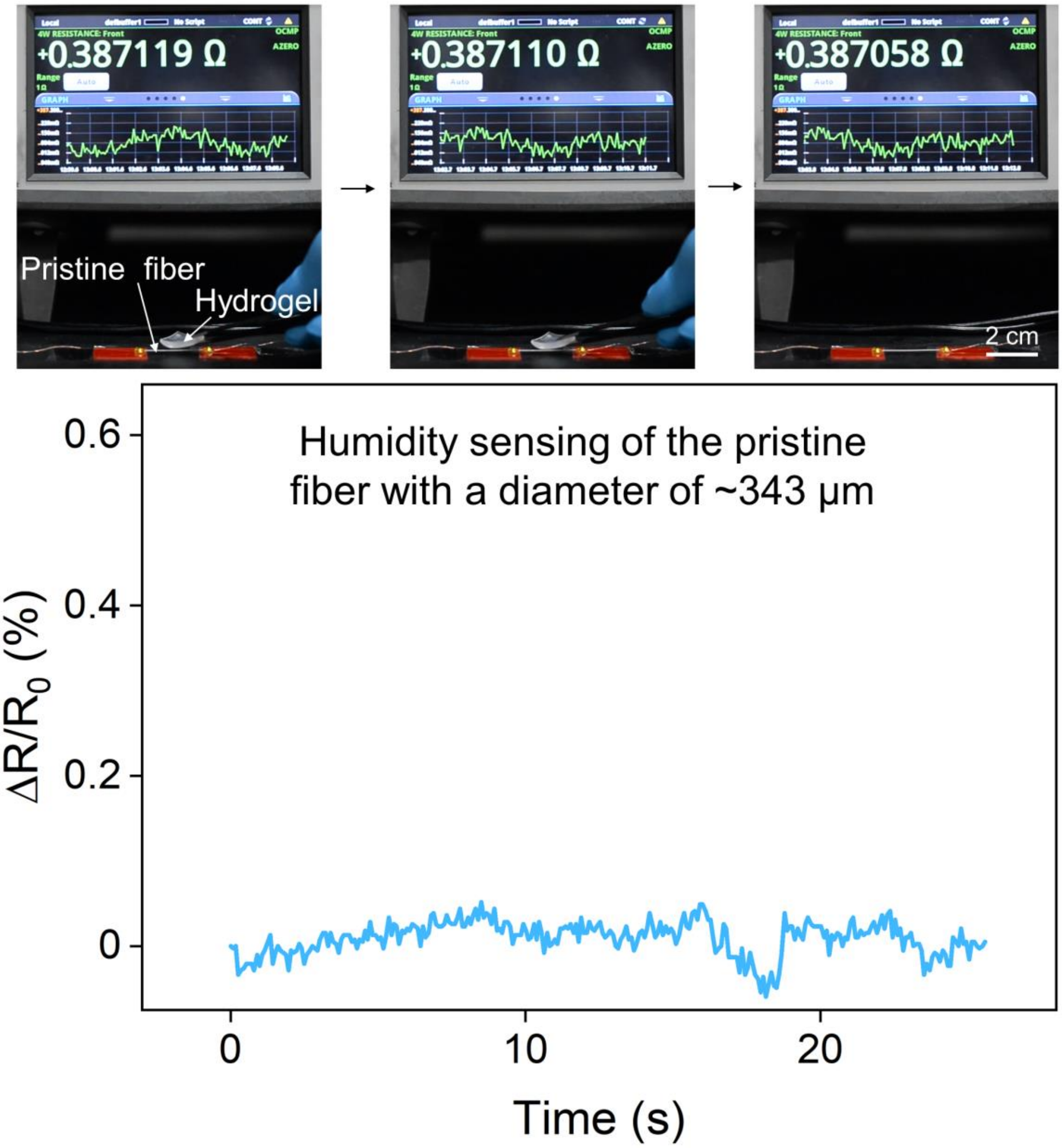

Figure S4. The pristine LM fiber with a diameter of ~343 μm showed no response to the humidity change of the environment.
